# Digital and virtual strategies to advance community stakeholder engagement in research during COVID-19 pandemic

**DOI:** 10.1017/cts.2022.457

**Published:** 2022-08-30

**Authors:** Samuel Byiringiro, Cyd Lacanienta, Roger Clark, Crystal Evans, Sarah Stevens, Melanie Reese, Pamela Ouyang, Mia Terkowitz, Christine Weston, Panagis Galiatsatos, Monica Guerrero Vazquez, Frederick W. Luthardt, Cheryl R. Dennison Himmelfarb

**Affiliations:** 1 Johns Hopkins School of Nursing, Baltimore, MD, USA; 2 Johns Hopkins Institute for Clinical & Translational Research, Baltimore, MD, USA; 3 Community Research Advisory Council, Baltimore, MD, USA; 4 Johns Hopkins University School of Medicine, Baltimore, MD, USA; 5 Office of Diversity, Inclusion, and Health Equity, Baltimore, MD, USA; 6 Center for Health and Opportunity for Latinos, Baltimore, MD, USA

**Keywords:** Community engagement, community-engaged research, virtual engagement, community stakeholders, COVID-19

## Abstract

Despite the adversity presented by COVID-19 pandemic, it also pushed for experimenting with innovative strategies for community engagement. The Community Research Advisory Council (C-RAC) at Johns Hopkins University (JHU), is an initiative to promote community engagement in research. COVID-19 rendered it impossible for C-RAC to conduct its meetings all of which have historically been in person. We describe the experience of advancing the work of the C-RAC during COVID-19 using digital and virtual strategies. Since March 2020, C-RAC transitioned from in person to virtual meetings. The needs assessment was conducted among C-RAC members, and individualized solutions provided for a successful virtual engagement. The usual working schedule was altered to respond to COVID-19 and promote community engaged research. Attendance to C-RAC meetings before and after the transition to virtual operation increased from 69% to 76% among C-RAC members from the community. In addition, the C-RAC launched new initiatives and in eighteen months since January 2020, it conducted 50 highly rated research reviews for 20 research teams. The experience of the C-RAC demonstrates that when community needs are assessed and addressed, and technical support is provided, digital strategies can lead to greater community collaborations.

## Introduction

*“Out of adversity comes opportunity*” Benjamin Franklin

The Coronavirus Disease 2019 (COVID-19) pandemic has had profound impacts on our society. In addition to claiming over 6 million lives as of May 2022, COVID-19 disrupted the usual channels of community engagement during a time when a trusting relationship between community and scientists was crucial for curbing the pandemic. The advances in communication technology, notably the ubiquity of social media, have fueled the transmission of conspiracy theories and misinformation, which play a harmful role in vaccine hesitancy, a current roadblock to stopping the COVID-19 pandemic [1]. The same advances in technology, however, can be effective in building new and maintaining existing trusting community partnerships to respond and curb the spread of COVID-19. Although virtual strategies for engaging the community were first proposed in 2017 [2], these strategies had not yet been implemented until COVID-19 pushed scientists and communities beyond their comfort zones.

The use of digital and virtual strategies for stakeholder engagement has seen its peak across different sectors of society. Zoom, a virtual meeting platform, saw the rise of daily meeting participants from 10 million in 2019 to 350 million by December 2020 [3]. The virtual and other remote strategies of engagement are some of the best possible means of maintaining and creating new trusting relationships with the community while minimizing social gatherings as required to prevent the spread of COVID-19 [4–6]. Collaboration with the community on these platforms provides an avenue to learn about community needs, share scientific information, and plan/implement research and projects that address those needs.

Community engagement in research has been proposed as a means of building trusting relationships between the community and scientists [7,8]. This concept embraces having community members involved in the entire process of the research continuum from ideation of research questions to the dissemination of findings. Community-engaged research (CEnR) offers a way forward to develop research programs which translate into policy and practice and provide solutions that are well received by the community [7,9]. Conducting community engagement by virtual means comes with opportunities and challenges. Some of the opportunities include providing a platform to enhance equity in access to research. For instance, the Hopkins Opportunities for Participant Engagement (HOPE), a registry developed by the Johns Hopkins Institute for Clinical and Translational Research (ICTR) promotes transparent and equal community participation in COVID-19-related research [10].

While discussing the possibility of virtual community engagement, it is important to understand and address the existing challenges. First, the community is diverse and has disparate access to technology resources such as computers, tablets, smartphones, and high-speed internet. There is additionally disparate level of knowledge, understanding, skills, and familiarity with technology and virtual engagement platforms (such as video conference software). According to the 2021 US Census Bureau report, 20% of the US population have no access to either broadband internet or a computer [11] and in Baltimore, 40.7% have no access to wireline internet (cable, fiber, or digital subscriber line service) and one in three homes lack a computer (desktop or laptop) [12]. The digital divide is the gap between people with and without computer and/or computer-like devices (smartphones) and internet access [13]. Lastly, over the last few decades, virtual engagement has been regarded as an ineffective means of community engagement compared with in-person engagement which in part explains why it was less explored/promoted [2]. The listed challenges affect some community members to a greater extent than others, especially those living in remote and underdeveloped neighborhoods, and individuals disadvantaged by socio-economic conditions.

To effectively engage the community in research using virtual and digital platforms, it is essential to conduct a thorough needs assessment followed by the response to the identified needs. In the current paper, we present an example of how the Community Research Advisory Council (C-RAC) at the Johns Hopkins ICTR continued community stakeholder engagement by transitioning to and supporting the use of virtual and digital platforms during COVID-19 and the resulting outcomes, despite the challenges listed.

### About Community Research Advisory Council

The Community Research Advisory Council (C-RAC) is part of the Johns Hopkins ICTR. First established in 2009, the C-RAC’s purpose is to promote trust, understanding, and involvement of the Greater Baltimore-Washington region in research, education, and service activities to enhance overall health, reduce health disparities, and promote social justice [14]. C-RAC membership is diverse, involving leaders and members of the community without Johns Hopkins affiliation here referred to as community members, and JHU faculty, staff, and students, here referred to as affiliates. To support that the C-RAC fully incorporates the community’s perspective, the C-RAC’s bylaws require that at least 50% of the members be community members.

C-RAC’s activities include the C-RAC’s flagship service “research consultation” through which C-RAC strives to ensure that the ‘real world’ perspectives of patients, community members, and diverse stakeholders are integrated into research at all stages of the research process. Researchers who wish to incorporate the community perspective in their research design and implementation deliberately (though sometimes this is as a requirement of research funders) request a consultation with the C-RAC. In addition to presenting and receiving community perspectives on their research, some researchers request letters of support for their grant proposal.

### C-RAC’s Transition to the Virtual Platform

Beginning in March 2020, C-RAC transitioned from in-person to virtual meetings responding to the public health mandates on social gatherings from the state and the government during the fight against the spread of COVID-19. Additionally, the sharing of information and materials such as handouts, moved online. To proactively address the digital divide prevalent in our communities, the ICTR’s staff contacted each C-RAC member and assessed the following: (1) how each member was faring in the pandemic, (2) member’s continued interest in meeting virtually, (3) the equipment capacity (computer, tablet or smartphone), (4) the current internet capacity (internet in the household/stability and speed of internet), (5) the need for customized technical assistance in navigating connectivity to virtual meetings and platforms.

Based on the findings of disparate access to tech devices for virtual meetings, internet connectivity, technical skills, and confidence to navigate the online resources and platforms, each member was provided customized solutions. The solutions included the ordering of Tablets for those who required them for virtual engagement and provision of one-on-one technical support. By the end of the transition period (April to May 2020), three tablets had been distributed, over a dozen sessions of individualized assistance were offered, IT virtual office hours for consults were made available. Assistance included one-on-one training on accessing C-RAC’s OneDrive, use of tablets, or smartphone devices, navigating Zoom functions, and opening electronically sent files.

From May 2020, the C-RAC initiated the COVID-19 expedited Community-Engaged Research Consultations to help respond to the COVID-19 pandemic. The expedited consultations consisted of a 1-hour meeting, conducted weekly, wherein the C-RAC provided a community perspective to researchers on their research aiming to address the COVID-19 pandemic. Johns Hopkins ICTR made available the budget to compensate community members for the increased frequency of meetings. Additionally, the C-RAC assisted investigators with ongoing studies looking for community advice on how to effectively integrate the new measures of social distancing and prevention of COVID-19 transmission.

### Evaluation Strategies

In our evaluation of the effectiveness of transition to virtual communications, we measured some of the contextual factors and group dynamics aspects of the conceptual logic model of community-based participatory research [15]. We tracked the attendance at the C-RAC meetings stratified by JH affiliation status as a process measure for community participation. We measured the outputs of the community-academic collaboration in terms of meaningful community contribution to the design of research projects. To do so, we tracked the number of new initiatives and partnerships born while operating remotely, the research consultations conducted while engaging with researchers remotely, and the letters of support provided to researchers applying for funding from January 2020 to June 2021.

In addition to tracking the number of research consultations, we collected the researchers’ feedback and satisfaction on the consultation services received during that same period via a 3–5-minute survey. The survey included questions on a 5-point Likert Scale from “strongly agree” to “strongly disagree” about how helpful the consultation was to them and open-ended questions about how areas for improvement. Researchers were also asked to rate their level of satisfaction with C-RAC consultation on the same scale from “very satisfied” to “very dissatisfied.”

## Results

### Attendance at the C-RAC Meetings

**Fig. 1.**
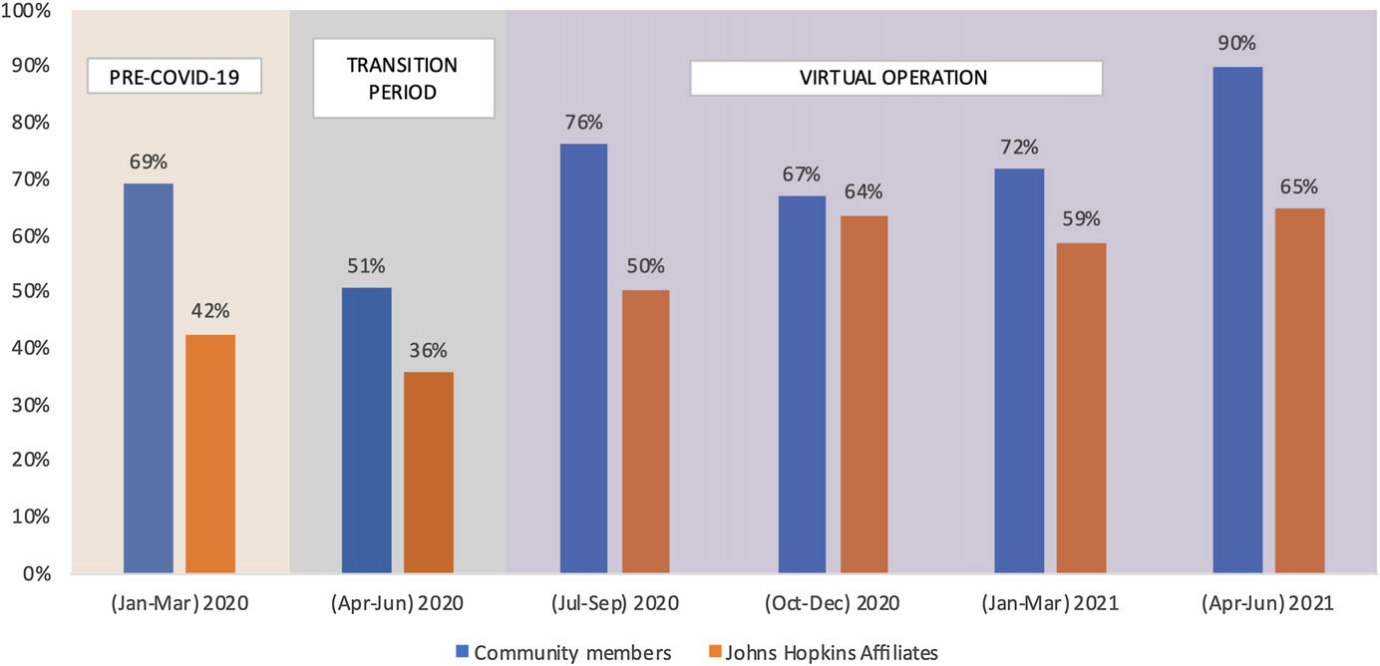
Attendance to Community Research Advisory Council (C-RAC) Meetings (Jan 2020–Jun 2021).

The transition to virtual meetings and provided resources and technical assistance improved participation among members. The attendance to in-person C-RAC meetings from January to March 2020 (pre-COVID-19) was 69% and 42% for community members and affiliates, respectively (Fig. 1). The attendance to meetings plunged lowest during the period of transition from in-person to virtual operation from April to June 2020 (51% and 36% among community members and affiliates respectively). The attendance to virtual meetings jumped higher than the in-person meetings from July 2020 onwards and peaked in April to June 2021 where the attendance was 90% and 65% among community members and affiliates, respectively. At all times, the attendance of C-RAC community members was higher than the affiliates.

### C-RAC Response to COVID-19 – a New Initiative

**Fig. 2.**
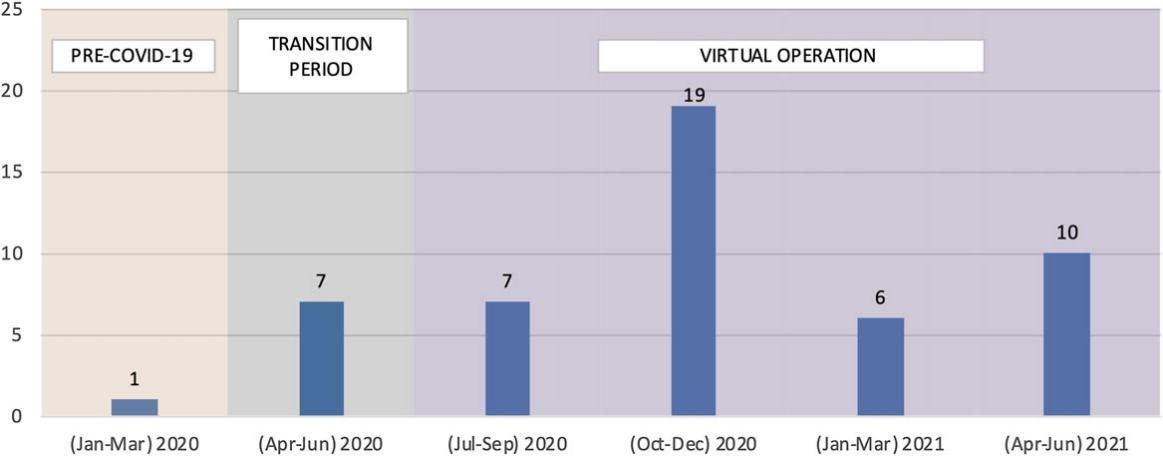
Number of Research Reviews Conducted (Jan 2020–June 2021), Total reviews = 50.

Improved virtual participation was associated with an increase in C-RAC’s consults. The C-RAC conducted a total of 50 research reviews (34 research reviews in 2020 and 16 reviews from January to June 2021) for 20 research teams including two Translational Science Linked Training (TL1) Scholars’ teams (Fig. 2). Among all the teams that C-RAC assisted, eight were conducting research to address COVID-19 pandemic, and four sought assistance on re-designing their studies to adhere to COVID-19 social distancing restrictions.

Research teams were encouraged to return and provide updates on their study or funding application or seek additional support if needed. Two teams presented four times during the period of the current analysis, four teams presented twice, and the rest have presented once, to date.

### Training a Future Generation of Researchers Who Uphold Community Engagement – A New Partnership

Transition to virtual meetings also offered the opportunity to expand C-RAC consults to other programs. While operating remotely, C-RAC initiated a partnership with the Clinical and Translational Science Scholars Program (TL1 scholars). In this partnership, TL1 scholars presented their research proposal to the C-RAC, received feedback on the design, implementation, and dissemination of their research, and returned to present the findings of their research. The methods and outcomes of this partnership have been well described [16]. Among the 50 research reviews conducted between January 2020 and June 2021, 24 were performed for TL1 scholars.

### Letters of Support for Grant Application

While collaborating remotely, C-RAC provided nine letters of support to investigators applying for research funding. Two of the nine applications directly related to addressing the COVID-19 pandemic while the remaining seven were addressing other challenges of healthcare access and complications caused by COVID-19 pandemic.

### Researchers’ Feedback to the C-RAC Research Consultation

**Fig. 3.**
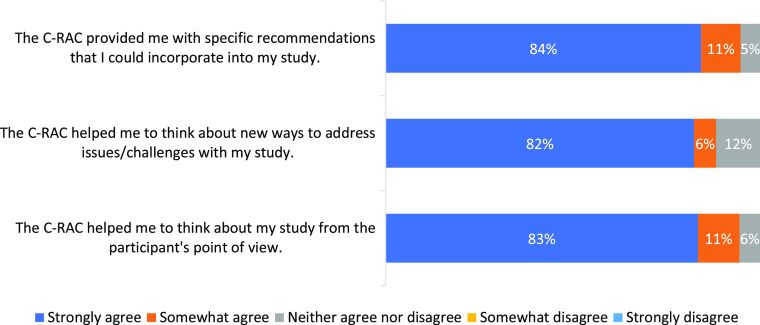
Researchers’ perception of Community Research Advisory Council (C-RAC) Research Reviews (*N* = 21).

**Fig. 4.**
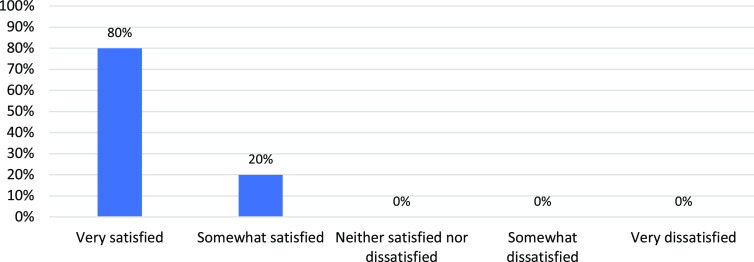
Researcher satisfaction with the Community Research Advisory Council (C-RAC) Research Review (*N* = 20).

The feedback from researchers who use the C-RAC consultation services was overwhelmingly positive. Twenty-one out of 50 total reviews received post-review researcher feedback. When asked if the C-RAC provided them with specific recommendations that they could incorporate into their study, 95% responded strongly or somewhat agree (Fig. 3). When asked whether the C-RAC had helped them think about new ways to address issues and challenges with their study, 88% replied strongly or somewhat agree. When asked if the C-RAC had helped them think about their study from the participant’s perspective, 94% responded strongly or somewhat agree. On the above three statements, 10% or less responded unsure (neither agree or disagree). Overall, 100% of the 20 respondents were very or somewhat satisfied with the consultation they received (Fig. 4).

## Discussion

The C-RAC at JHU has been very successful in not only keeping up with its work, but also in increasing the number of consults and expanding the scope of its services, despite the numerous disruptions imposed by COVID-19 pandemic. The successful continuity of operation is the result of the community achieving a quick transition to virtual and remote operation. This transition was achieved after a community virtual operation needs assessment was performed and individualized support was provided. The provision of technical assistance not only increased participation but also sustained participation over time. Most community members quickly became fluent with remote operation. New initiatives and partnerships were launched, and researchers provided overwhelmingly positive feedback on the services offered by the C-RAC.

The transition to virtual operation increased stakeholder engagement and participation through online meetings. As a result of more frequent meetings, the C-RAC conducted 50 research reviews in the period of 18 months while operating remotely as compared to 30 research reviews conducted within 10 years (2009–2019). These findings are consistent with the report of the virtual program developed by Galiatsatos and colleagues [17], who within a period of 2 months, conducted 12 community calls of approximately 125 participants each with community members, leaders of community-based organizations and religious leaders during COVID-19 pandemic. The stakeholders in many other community engagement partnerships locally in the United States, in other countries, and across countries and continents commended the openness to virtual engagement which became more apparent during COVID-19 pandemic [18–21]. Some of the additional benefits of virtual engagement pointed out by those stakeholders are the flexibility, avoidance of travel costs, bypassing geographical inaccessibility, reducing travel burden for people with physical disabilities, and options to record the meetings for people who cannot attend synchronically.

The attendance to C-RAC meetings remained higher among community members than JHU affiliates’ during the entire period of the current analysis. Given the university resources made available to JHU affiliates and the fact that the similar difference was present prior to virtual transition, we are not certain as to whether that difference has anything to do with adjustment to virtual engagement or digital divide. It has come to light that the digital divide goes beyond access to tech devices and internet but rather to the ability to: get proper maintenance of the devices; afford the cost of the internet; receive training to navigate and utilize the technology; and have access to technical support [22]. Despite the ubiquity of technology at the universities, some faculty, staff and students especially the older generation, lack the expertise and fluency in navigating the technology yet shy away from seeking support. A systematic review exploring students and university teachers’ digital competence found basic level of competence [23]. Another possible reason for disparities in attendance to meetings could result from the fact that the community members but not JHU affiliates receive compensation for their attendance to C-RAC meetings. Some C-RAC members speculated that faculty, staff, and students bear additional responsibilities which could impact their attendance to C-RAC activities, however a separate study is proposed to assess the factors influencing high or low commitment by community members and academic affiliates to community–academic partnerships.

The community–academic partnerships are some of the collaborations encouraged to promote community trust in science and advance the fight against COVID-19. It is important to note that while first responders are the obvious heroes in the fight against COVID-19, the community members such as C-RAC members are the unsung heroes who dedicated more of their time to learn the unconventional means of engagement (virtual strategies) and use them to ensure that the research aiming to address COVID-19 challenges gets the right participants and is well received by the community. C-RAC reviewed studies that early on promoted COVID-19 screening uptake, then COVID-19 vaccine uptake, and general trust in science to name a few. Clearly, virtual engagement has great potential to transform ways in which community informs the research, engages in research, and takes action synergistically with in-person meetings and in times when in-person interactions are not an option.

Despite the many benefits associated with virtual community engagement in research, any advances made may quickly be jeopardized because of the aforementioned barriers including digital divide. Our success in transitioning to virtual and digital operation might stem essentially from the needs assessment, provision of tech devices, internet access, and the individualized support that were rendered readily available; yet, we still have not figured out the strategy to sustain the cost of internet access for members moving away from the pandemic. Supporting city-wide solutions on internet connectivity in all neighborhoods should be a top priority systemically. Bridging the digital divide gap and increasing community members’ capacity on virtual engagement would not only maintain engagement but also address structural inequality in participation in research and accessing healthcare experienced by many community members especially the Hispanics and Black community.

## Conclusion

The current paper highlights the success story of the C-RAC transitioning from in-person interactions and engagement to virtual operation during COVID-19 pandemic. The adverse situation imposed by COVID-19 pushed the group to realize that digital and virtual strategies are an additional effective method for engaging with community as stakeholders and partners in research. Going forward, it is essential to consider virtual and digital strategy as an opportunity to address historical challenges to community engagement such as geographic inaccessibility. Yet, community investment (access to tech devices, internet, knowledge, and support) is required for the virtual and digital engagement opportunity to be equally accessible by all community members. If community needs are assessed and technical support provided, digital strategies can lead to stronger partnership with the community and greater collaboration as was demonstrated by the C-RAC experience.
